# Occupational Rehabilitation Is Associated With Improvements in Cognitive Functioning

**DOI:** 10.3389/fpsyg.2019.02233

**Published:** 2019-10-10

**Authors:** Thomas Johansen, Chris Jensen, Hege R. Eriksen, Peter S. Lyby, Winand H. Dittrich, Inge N. Holsen, Hanne Jakobsen, Irene Øyeflaten

**Affiliations:** ^1^National Advisory Unit on Occupational Rehabilitation, Rauland, Norway; ^2^Department of Public Health and Nursing, Norwegian University of Science and Technology, Trondheim, Norway; ^3^Department of Sport, Food and Natural Sciences, Western Norway University of Applied Sciences, Bergen, Norway; ^4^CatoSenteret Rehabilitation Center, Son, Norway; ^5^FOM Hochschule, KCI Competence Center for Behavioral Economics, Frankfurt, Germany; ^6^Red Cross Haugland Rehabilitation Center, Flekke, Norway; ^7^Valnesfjord Health Sports Center, Fauske, Norway; ^8^Norwegian Research Centre, Bergen, Norway

**Keywords:** occupational rehabilitation, cognition, attention, work ability, sick leave

## Abstract

**Introduction:**

Occupational rehabilitation may be offered to workers on long-term sick leave who often report problems with cognitive functioning, anxiety, depression, pain, and reduced work ability. The empirical knowledge is sparce on how occupational rehabilitation may influence cognitive and emotional functioning and patients have not previously been subjected to comprehensive objective testing. The main aim of this study was to assess possible changes in cognitive and emotional functioning such as memory, attention, executive function, and emotion recognition among patients in occupational rehabilitation.

**Methods:**

A large sample of 280 sick-listed workers referred to inpatient and outpatient occupational rehabilitation was recruited. The rehabilitation programs had a mean duration of 28 days and comprised physical activity, cognitive behavior treatment components and collaboration with the workplace. A pre–post design was applied to investigate possible changes in cognitive and emotional functioning (primary outcomes) and work and health measures (secondary outcomes), comparing the rehabilitation group with a control group of 70 healthy workers. Individuals in the control group were tested at random time points with an approximately 28 day interval between pre- and post-test, thus coinciding with the duration of rehabilitation. Repeated measures analysis of variance was used for the main analyses.

**Results:**

Compared to the control group, the rehabilitation group had greater gains from pre- to post-test in focused and sustained attention, as well as greater improvements in work ability and reduction in subjective health complaints (SHC), helplessness, pain, pain related to work, anxiety, and depression. In the rehabilitation group, exploratory correlational analysis indicated that improvements in focused and sustained attention were associated with improvements in return-to-work self-efficacy, work ability as well as a reduction in SHC.

**Conclusion:**

The sick-listed workers improved in focused and sustained attention and work and health measures after participating in occupational rehabilitation. This study is one of the first to systematically investigate changes in cognitive and emotional functioning during occupational rehabilitation. Clinical practice should benefit from increased knowledge about all cognitive functions and should be specifically aware of the improvements in focused and sustained attention, while memory, executive function and emotion recognition remained unchanged. The results can be used as a motivation to tailor specific interventions to gain further improvements in all cognitive and emotional functions.

## Introduction

Workers on long-term sick leave referred to occupational rehabilitation report memory and attention problems, symptoms of anxiety, depression and pain, and reduced work ability (Aasvik et al., 2015; Johansen et al., 2016; Aasdahl et al., 2017). These factors are assumed to negatively affect the ability to concentrate on work tasks, process information and shift attention when required in working life. On the other hand, well-preserved cognitive functioning improves flexibility and the capacity to regulate our thoughts, emotions and behavior (Dajani and Uddin, 2015). Understanding how occupational rehabilitation affects cognitive functioning, work ability and health related factors is important because it can be assumed that improvements in these measures contribute positively to return to work (RTW) (Eskildsen et al., 2016; Johansen et al., 2016). However, the investigation of changes in cognitive functioning during occupational rehabilitation has so far been an understudied topic relying only on preliminary evidence (Johansen et al., 2016; Aasvik et al., 2017). This is a highly relevant topic because anxiety, depression and pain are associated with impairments in cognitive functioning (Yiend, 2010; Landrø et al., 2013; Snyder et al., 2015) and because maintaining good cognitive processing is an important premise for adaptive emotion regulation (Ochsner and Gross, 2005). If cognitive impairments in attention, memory and executive function are present in workers on long-term sick leave (Eskildsen et al., 2015, 2016; Johansen et al., 2016) it is relevant to assess if improvements in cognitive functioning occur during occupational rehabilitation and whether these improvements are associated with work ability, anxiety, depression and subjective health complaints (SHC).

In Norway, the rehabilitation programs often consist of work-related, physical and cognitive behavior treatment components (Øyeflaten et al., 2016). These components include adapted physical activity as well as a cognitive approach based on principles from cognitive behavior therapy, acceptance and commitment therapy, psychoeducation and motivational interviewing. The positive effects of physical activity on cognitive functioning (Ratey and Loehr, 2011), anxiety and depression (Hovland et al., 2013; Kvam et al., 2016) are well documented. Collaboration with the employer, the general practitioner and the social security offices is also a key component (Fimland et al., 2014; National Advisory Unit on Occupational Rehabilitation, 2017). During rehabilitation, there is a strong emphasis on improving patients’ work ability, self-efficacy, and RTW expectation.

Similar patient populations to those being referred to occupational rehabilitation have been subjected to cognitive testing. Eskildsen et al. (2015, 2016) retested a group of patients on sick leave due to work-related stress 1 year after being referred to occupational and psychological therapy. It was found that patients improved more in prospective memory and processing speed compared to a healthy control group. In two separate studies, conducting pre-test and 1 year follow up assessment, it was reported that women sick-listed for work-related stress and depression, and receiving 10 weeks of cognitive group therapy addressing work-related challenges, improved in depressive symptoms as well as in cognitive functioning compared to a control group (Rydmark et al., 2006; Wahlberg et al., 2009). On a general note, it can be assumed that impairments in attention, memory and executive function might contribute to symptoms of anxiety, depression and pain, however, it could also be argued that symptoms of anxiety, depression and pain are associated with impairments in cognitive functioning. There seems to be a debate about the direction of influence (Legrain et al., 2009; Yiend, 2010; Snyder et al., 2015). The two positions are not mutually exclusive and may differ depending on individual differences, health status and type of cognitive or executive impairment (Snyder et al., 2015).

Following previous studies reporting on changes in cognitive functioning (Eskildsen et al., 2015, 2016; Johansen et al., 2016; Aasvik et al., 2017), it was important in the current study to analyse a broad spectrum of functions such as attention, memory, executive function and emotion using validated and computerized cognitive and emotional tests. In addition, the recognition of facial expressions was measured as cognitive and emotional processing is strongly related (Yiend, 2010; Pessoa, 2013). For example, individuals reporting anxiety seem to have an attentional bias toward fear, while depression is related to a bias toward sadness (Yiend, 2010). The aim of the current study was to compare possible changes in cognitive and emotional functioning and work and health measures between sick-listed workers and healthy workers, and to explore the degree of association between changes in cognitive and emotional functioning and work and health measures.

## Materials and Methods

### Participants

A total of 280 patients who were either on partial or full sick leave, volunteered to take part in the study. 187 participated in an inpatient occupational rehabilitation program and 93 in an outpatient program. Seventy workers in the control group who volunteered to take part were all working full time and had no sick leave during the testing period. They were recruited from the wider community and employees from three rehabilitation clinics and included a wide selection of different blue- and white-collar workers. The two groups were matched for age, gender and number of days between pre- and post-test. 80% of the patients had an ICD-10 diagnosis either in the categories *F*, mental and behavioral disorders or M, diseases of the musculoskeletal system and connective tissue. Exclusion criteria for the rehabilitation and control group were a history of head injury or having applied for disability pension.

### Design

This study had a non-randomized pre–post measures controlled design. All participants in the study were assessed with cognitive and emotional tests and work and health questionnaires. The study was designed to analyse differences between-subjects (intervention vs. control group) and within-subjects (cognitive and emotional tests and questionnaires at pre- and post-test).

### Occupational Rehabilitation

The patients were referred to occupational rehabilitation by general practitioners or social security offices. The main aim of rehabilitation was RTW and the programs lasted between three and 12 weeks. The patients were followed up by an interdisciplinary team including at least four of the following professionals: physician, physiotherapist, psychologist, work consultant, coach, nurse/psychiatric nurse, and sports pedagogue. Assessment of work ability, physical fitness and current work and health situation was carried out to tailor rehabilitation efforts. Key interventions were adapted physical activity, cognitive behavior treatment components and collaboration with the workplace, general practitioner and social security office. Adapted physical activity included supervised exercise individually adjusted to needs and physical capacity. Exercise types included ergometer cycling, outdoor walking, resistance exercise, and enduring exercise. The cognitive behavior treatment components focusing on work and health included principles based on cognitive behavior therapy and psychoeducation for anxiety, depression, pain; adapted physical activity and the effects on body and mind; behavioral activation relevant for depression; skills training in problem solving; mindfulness; pain education; fear avoidance beliefs and exposure principles at work and during physical activity.

### Norwegian Sickness Insurance

Individuals who are unable to work due to illness or injury are entitled to sick leave benefits from the Norwegian sickness insurance scheme for a maximum of 52 weeks. For the first 16 days, full compensation is provided by the employer and thereafter by the tax-paid national insurance system. If the individual is still unable to resume partial or full-time work after 1 year, a work ability assessment will determine if further benefits for up to 3 years may be granted. The benefits after the first year are normally two thirds of the wages the individual had prior to sick leave. The benefits can be combined with partial work resumption.

### Outcome Measures

The primary outcome measures were performance changes from pre- to post-test on the cognitive and emotional tests targeting attention, memory, executive function and emotion. The secondary outcome measures were changes from pre- to post-test on the questionnaires targeting work and health characteristics.

### Materials

#### Cognitive and Emotional Tests (Primary Outcomes)

Eight validated tests from the Cambridge Neuropsychological Test Automated Battery (CANTAB) were used to assess cognitive and emotional functioning on a touch screen (Table 1). The order of the tests was fully counterbalanced across participants at each testing session and within each group. All participants were introduced to the touch screen by way of a motor screening task performed prior to testing both at pre- and post-test. This screening was performed to familiarize the participants with the touch screen and reduce as much as possible any initial apprehension prior to testing.

**TABLE 1:** Description of computerized cognitive and emotional tests.

**Test**	**Description**	**Outcome variables**	**Cognitive function**
Simple reaction time	Participants must press as fast as they can a button on a press pad when they see a white square in the middle of a computer screen. The task consists of one block of 24 practice trials and two assessment blocks each with 50 trials	Mean correct reaction time	Focused attention
Choice reaction time	Participants must press as fast as they can the left hand button on a press pad when they see an arrow pointing to the left and press the right hand button when an arrow is pointing to the right. The task consists of a block of 24 practice trials and two assessment blocks each with 50 trials	Mean correct reaction time	Selective attention
Rapid visual information processing	Participants have to detect and respond by pressing a button on a press pad to target sequences 3-5-7, 2-4-6, and 4-6-8, from digits between 2 and 9 appearing one at a time in a pseudo-random order lasting 4 min	Mean correct response latency, probability of hits	Sustained attention
Spatial working memory	The participants must search for blue tokens hidden inside boxes and the trial is completed when a token has been found in each box. Four practice trials are given each with three boxes and the assessed trials include two blocks of four, six, and eight boxes	Mean number of between errors (revisiting boxes which have already been found to contain a token)	Visuospatial working memory
Spatial recognition memory	In the presentation phase five white squares appear one at a time in different locations on a computer screen and then in the assessed stage the participant is presented with two squares in different locations and must touch the square that was in the correct location in the presentation phase. The task consists of four blocks each with five new locations	Mean correct response latency, mean percentage correct	Visuospatial recognition memory and short term memory
Stockings of Cambridge	Participants are asked to copy a predetermined arrangement of balls. Only one ball can be moved at a time to an empty pocket or on top of another ball. The aim is to use the minimum number of moves required to solve each problem (2 × 2 move, 2 × 3 move, 4 × 4 move and 4 × 5 move)	Mean choice duration, mean number of problems completed in the minimum number of moves (maximum 12)	Executive spatial planning and spatial working memory
Intra-extra dimensional set shift	Participants are shown two stimuli and must touch the correct one taking into account feedback provided on each trial and can thus learn which one is correct. The task consists of nine stages and to pass each stage a criterion of six consecutive correct responses is required	Mean number of trials to reach criterion at the extradimensional shift stage (stage 8; attentional shifting away from a previously relevant stimulus dimension)	Attentional shifting and flexibility
Emotion recognition task	A face showing either happiness, sadness, anger, fear, disgust or surprise is shown and participants must touch the emotion they believe is correct by selecting one of six written emotions on the screen directly after each face disappear. Each facial emotion has 15 different levels from hardly any emotional expression to a clear expression and they see all emotions once. The task consists of one block of 90 faces	Mean percentage correct	Emotion and social cognition

#### Work and Health Questionnaires (Secondary Outcomes)

The participants in the control group completed all questionnaires except Return to Work Self-Efficacy (RTWSE-19 items; Shaw et al., 2011; Nøttingnes et al., 2019). Work ability was assessed using one item from the work ability index comparing current work ability with the lifetime best (Ahlstrøm et al., 2010); RTWSE-19 (Shaw et al., 2011) assessed the participants’ belief in their own ability to resume normal work tasks according to the following factors: meeting job demands, modify job tasks and communicating needs to others; The importance of performing well on the cognitive tests was assessed on a five-point rating scale using a newly developed item by the project group (“To what degree is it important for you to perform well on the cognitive tests?”); Two subscales from the SHC inventory (Eriksen et al., 1999) assessed participants’ health complaints during the last 30 days according to pseudoneurology (extra heartbeats, heat sensation, sleep problems, tiredness, dizziness, anxiety, sad/depression) and musculoskeletal pain (headache, neck pain, upper back pain, low back pain, arm pain, shoulder pain, migraine, leg pain during physical activity); Theoretically Originated Measure of the Cognitive Activation Theory of Stress (TOMCATS; Odeen et al., 2012) assessed positive, negative and no response outcome expectancies i.e., one item for coping, three items for hopelessness and three items for helplessness; the Fear Avoidance Beliefs Questionnaire (FABQ; Waddell et al., 1993) measured avoidance beliefs for physical activity (four items) and work (seven items), on separate scales; Items seven and eight from the short form 36 health survey (SF-36; Ware and Sherbourne, 1992) assessed pain and pain related to work respectively; the Hospital Anxiety and Depression Scale (HADS; Zigmond and Snaith, 1983) covered seven items for symptoms of anxiety and seven items for symptoms of depression.

All measures described above are validated except the single items pain and pain related to work from SF-36 and “to what degree is it important for you to perform well on the cognitive tests?”

### Statistical Analyses

Data were analyzed using SPSS version 25 (SPSS Inc., 2019). The categorical variables gender and education were compared between the intervention and control group using chi-square analysis and the variable age was subjected to independent samples *t*-test. Demographic, work and health measures at baseline were analyzed with independent samples *t*-tests. To investigate changes in cognitive and emotional functioning and work and health measures from pre- to post-test a repeated-measures analysis of variance was conducted using a 2 × 2 mixed design with group (intervention, control) as a between-subjects factor and time (pre-test, post-test) as a within-subjects factor. The interaction effects were tested using the multivariate criterion of Wilks’ lambda for all *F* tests to overcome the assumptions of univariate testing. Education was included as a covariate because it differed between the two groups and estimated marginal means are indicated. Significance threshold was *p* < 0.05. It was not adjusted for the cognitive tests since they are assumed to target separate cognitive processes having different ecological validity (Levine et al., 2011). If assumptions of homogeneity of variance and normality were not met, logarithmic (Base 10) transformations were performed (Tabachnick and Fidell, 2001). When data transformation procedures were undertaken it was decided to display the means and standard deviations for the untransformed scores for standardization and clarity purposes to enable comparison of performance data across different studies while the statistics for transformed scores were reported in the text and tables. Consequently, a logarithmic (Base 10) transformation was used on the following task measures in both groups; simple and choice reaction time, response latency for rapid visual information processing, response latency for spatial recognition memory and choice duration for stockings of Cambridge to reduce skewness and kurtosis in the distributions. To explore whether changes in cognitive, emotional, work and health measures correlated, the difference in the significant outcome variables between pre- and post-test was calculated in the intervention group only. The effect size measure for partial eta-squared (ANOVA) was interpreted according to the following values (Cohen, 1988): below 0.06 small, 0.06–0.14 moderate and above 0.14 large. For Pearson product-moment correlational coefficients the following values were used (Cohen, 1988): 0.10 small, 0.30 moderate, and 0.50 large.

*A priori* power calculations for *F* tests using G^∗^Power (Faul et al., 2007) were performed to check which sample size was required to detect differences between groups in scores on cognitive and emotional tests and work and health questionnaires using repeated-measures analysis of variance. Results indicated that with a power of 0.90, moderate effect size set at 0.10 and a two-tailed alpha level of 0.05, the total number of participants needed would be 58 in each group.

## Results

For the total sample, floor and ceiling effects on the cognitive and emotional measures for correct responses were considered low, ranging from 0.3 to 4.6%, except for between search errors in spatial working memory, where floor effects were 12% both at pre- and post-test, and number of problems solved in stockings of Cambridge, where ceiling effects were 9.7% at pre-test and 15.1% at post-test. On the reaction time variables for simple reaction time and choice reaction time, there were no false alarm scores indicating responses below 100 ms or above 1000 ms, and thus floor and ceiling effects were zero.

### Baseline Demographic, Work and Health Characteristics

Mean age was similar between the rehabilitation (mean 45.1, standard deviation 9.6) and control group (mean 46.6, standard deviation 9.7), while at baseline, level of education differed because more participants in the control group had completed higher education (Table 2). The primary diagnoses of the rehabilitation participants were according to ICD-10 musculoskeletal (53%) and mental and behavioral (27%) disorders, while all other diagnostic categories accounted for 20%.

**TABLE 2:** Demographic characteristics in the intervention and control group.

	**Intervention group**		**Control group**		
	**(*n* = 280)**		**(*n* = 70)**		**Difference**
	***n***	**%**	***n***	**%**	**X^2^(df)**
**Gender**					3.65 (1)
- Female	187	67	55	79	
- Male	93	33	15	21	
**Education**					35.31^∗∗∗^ (2)
- Elementary	38	14	1	1.5	
- Secondary	124	44	12	17	
- Higher	118	42	57	81.5	
**Work status**					Not relevant
- Not in work	161	57.5	0	0	
- Part time work	118	42	0	0	
- Full time work	1	0.5	70	100	
**Diagnostic codes from the ICD-10:**					
C. Malignant neoplasms	4	1.5			
E. Endocrine, nutritional and metabolic diseases	1	0.5			
F. Mental and behavioral disorders	73	27			
G. Diseases of the nervous system	21	7.5			
I. Diseases of the circulatory system	6	2			
J. Diseases of the respiratory system	1	0.5			
K. Diseases of the digestive system	1	0.5			
L. Diseases of the skin and subcutaneous tissue	1	0.5			
M. Diseases of the musculoskeletal system and connective tissue	144	53			
R. Symptoms, signs and abnormal clinical and laboratory findings, not elsewhere classified	4	1.5			
S. Injury, poisoning and certain other consequences of external causes	2	0.5			
Z. Factors influencing health status and contact with health services	14	5			

*Diagnostic codes applicable only to the intervention group (codes not retrievable for eight patients).*

Details about the range and scoring direction of each questionnaire can be seen in Table 3. Individuals in the rehabilitation group had on average been on partial or full sick leave for more than 6 months. The rehabilitation group found it more important to perform well on the computerized tests compared to the control group. The rehabilitation group scored lower than the control group in work ability and had higher levels of anxiety, depression, pain (SF-36 item seven), pain related to work (SF-36 item eight), fear avoidance for work, fear avoidance for physical activity, SHC pseudoneurology and SHC musculoskeletal pain. On the TOMCATS, the rehabilitation group scored worse than controls in coping, hopelessness and helplessness.

**TABLE 3:** Baseline characteristics for demographic, work and health measures for intervention and control group.

	**Intervention group (*n* = 280)**	**Control group (*n* = 70)**	**Difference**
	**Mean *(SD)***	**Mean *(SD)***	***T* (df^#^)**
Number of days between pre- and post-test	28.2 (23.3)	28.3 (6.1)	0.04 (364)
Full or part time sick leave prior to rehabilitation (months)	6.5 (4.1)	Not relevant	
Performance on computerized tests (range 1–5; 5 = most important)	2.9 (1.1)	2.5 (1.1)	2.32(314)^∗^
Work ability (range 0–10; 10 = best work ability)	3.8 (2.4)	8.8 (1.3)	15.86(326)^∗⁣∗∗^
Return-to-Work Self-Efficacy-19			
- Meeting job demands (range 1–70; 70 = highest self-efficacy)	34.0 (18.4)	Not relevant	
- Modifying job tasks (range 1–60; 60 = highest self-efficacy)	28.6 (13.2)	Not relevant	
- Communicating needs (range 1–60; 60 = highest self-efficacy)	36.2 (14.6)	Not relevant	
Subjective Health Complaints Inventory			
- Pseudoneurology (range 0–21; 21 = highest severity)	7.1 (4.0)	2.0 (2.5)	9.05(319)^∗⁣∗∗^
- Musculoskeletal pain (range 0–24; 24 = highest severity)	9.9 (5.0)	3.2 (3.5)	9.52(318)^∗⁣∗∗^
Theoretically Originated Measure of the Cognitive Activation Theory of Stress			
- Coping (range 1–4; 1 = best coping)	2.0 (0.6)	1.7 (0.6)	3.65(317)^∗⁣∗∗^
- Hopelessness (range 1–12; 1 = most hopelessness)	9.1 (1.9)	10.7 (1.8)	5.72(318)^∗⁣∗∗^
- Helplessness (range 1–12; 1 = most helplessness)	9.6 (2.0)	11.2 (1.6)	5.31(315)^∗⁣∗∗^
Fear Avoidance Beliefs Questionnaire			
- Work (range 0–42; 0 = no fear avoidance)	20.4 (11.5)	2.6 (5.6)	9.52(283)^∗⁣∗∗^
- Physical activity (range 0–24; 0 = no fear avoidance)	8.9 (5.9)	2.2 (4.3)	6.94(288)^∗⁣∗∗^
36-Item Short Form Health Survey			
- Pain (item seven) (range 1–6; 1 = no pain)	4.5 (1.1)	2.5 (1.3)	12.17(315)^∗⁣∗∗^
- Pain related to work (item eight) (range 1–5; 1 = not affected)	3.4 (1.1)	1.6 (0.9)	11.62(315)^∗⁣∗∗^
Hospital Anxiety and Depression Scale			
- Anxiety (range 0–21; 0 = no anxiety)	8.3 (4.4)	4.2 (3.6)	6.42(311)^∗⁣∗∗^
- Depression (range 0–21; 0 = no depression)	6.4 (4.0)	2.3 (2.9)	7.05(311)^∗⁣∗∗^

**n*, sample size; SD, standard deviation; *T*, T statistic; df, degrees of freedom. ^#^All participants did not respond to all questionnaire items or single item questions. ^∗^*p* < 0.05. ^∗∗∗^*p* < 0.001.*

### Changes in Cognitive Functioning

**FIGURE 1:**
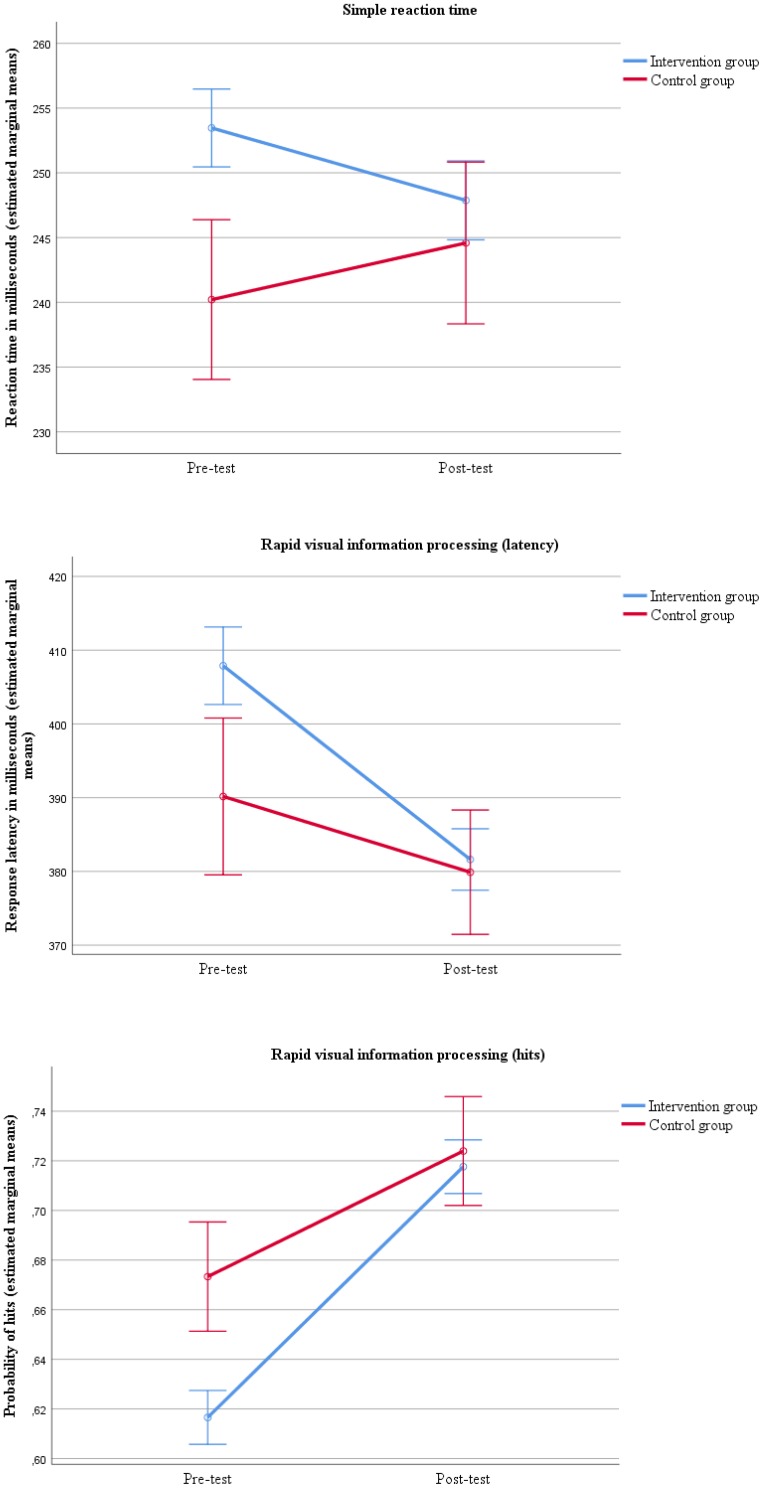
Improvement in attention from pre- to post-test comparing the intervention and control group with errors bars showing ±1 standard error.

The between group scores from pre- to post-test showed that the intervention group had greater improvements in reaction time on the simple reaction time test [Λ = 0.99, *F*(1, 345) = 3.93, *p* = 0.048] and in response latency [Λ = 0.99, *F*(1, 338) = 4.17, *p* = 0.042] and hits [Λ = 0.98, *F*(1, 339) = 6.43, *p* = 0.012] on the rapid visual information processing test compared to the control group (Figure 1 and Table 4).

**TABLE 4:** Pre- and post-test scores for intervention and control group on cognitive and emotional tests with group by time effect.

		**Intervention group (*n* = 280)**		**Control group (*n* = 70)**		**Group by time effect**		
**Test**	**Time**	**Mean (SD)**	**EMM**	**Mean (SD)**	**EMM**	***F* (df)**	***p***	**${\text{η}}_{\text{p}}^{2}$**
**Simple reaction time**								
Reaction time (ms)	Pre-test	253.6 (52.6)	253.5	239.8 (34.8)	240.2	3.93(1,345)	0.048	0.011
	Post-test	248.0 (52.1)	247.9	244.1 (41.9)	244.6			
**Choice reaction time**								
Reaction time (ms)	Pre-test	322.2 (65.1)	321.8	307.8 (39.2)	309.2	1.58(1,344)	0.210	0.005
	Post-test	312.0 (53.9)	311.5	305.8 (41.1)	307.9			
**Rapid visual information processing**								
Response latency (ms)	Pre-test	408.6 (89.6)	407.9	387.6 (67.8)	390.2	4.17(1,338)	0.042	0.012
	Post-test	381.6 (68.4)	381.6	379.8 (65.3)	379.9			
Probability of hits	Pre-test	0.61 (0.18)	0.62	0.70 (0.18)	0.67	6.43(1,339)	0.012	0.019
	Post-test	0.71 (0.18)	0.72	0.74 (0.17)	0.72			
**Spatial working memory**								
Total between errors	Pre-test	12.4 (9.8)	12.3	11.1 (9.1)	11.5	0.74(1,347)	0.392	0.002
	Post-test	10.5 (9.2)	10.4	10.0 (9.8)	10.6			
**Spatial recognition memory**								
Response latency (ms)	Pre-test	2726 (978)	2719	2467 (1077)	2495	3.24(1,347)	0.073	0.009
	Post-test	2240 (690)	2234	2138 (601)	2160			
Total correct (%)	Pre-test	80.6 (10.1)	80.6	81.4 (11.4)	81.4	0.03(1,347)	0.854	0.000
	Post-test	81.0 (10.8)	81.1	82.1 (9.8)	81.7			
**Stockings of Cambridge**								
Choice duration (ms)	Pre-test	4149 (2256)	4128	4213 (1954)	4297	1.85(1,346)	0.175	0.005
	Post-test	3600 (1697)	3608	3501 (1435)	3470			
Total correct	Pre-test	8.9 (2.0)	8.9	9.3 (1.7)	9.3	1.01(1,347)	0.315	0.003
	Post-test	9.7 (1.8)	9.7	9.8 (1.9)	9.8			
**Intra-extra dimensional set shift**								
Trials extradimensional shift stage	Pre-test	9.4 (9.2)	9.3	10.3 (9.8)	10.6	0.01(1,347)	0.961	0.000
	Post-test	6.1 (8.1)	5.9	6.7 (9.1)	7.2			
**Emotion recognition task**								
Total correct (%)	Pre-test	59.0 (10.0)	59.3	60.5 (8.7)	59.5	3.09(1,347)	0.079	0.009
	Post-test	62.0 (10.1)	62.2	62.0 (10.2)	60.8			

**n*, sample size; SD, standard deviation; EMM, estimated marginal means evaluated with covariate in model (Education); df, degrees of freedom; ${\text{η}}_{p}^{2}$, partial eta squared; The multivariate test statistic Wilks’ lambda was used to display the *F* ratio; The drop out rate for each cognitive and emotional outcome variable varied in the intervention group from *n* = 315 to 307 at pre-test to *n* = 280 to 276 at post-test. In the control group, 70 out of the 73 participants completing pre-test were able to come back and participate at post-test.*

### Changes in Work and Health Characteristics

Between group differences showed that the rehabilitation group had greater improvements compared to controls from pre- to post-test in work ability, reported more reduction in pseudoneurology and musculoskeletal pain (SHC), helplessness (TOMCATS), pain and pain related to work (SF-36 items seven and eight) and anxiety and depression (HADS) (Table 5).

**TABLE 5:** Pre- and post-test scores for intervention and control group on work and health measures with group by time effect.

		**Intervention group (*n* = 280)**		**Control group (*n* = 70)**		**Group by time effect**		
**Variable**	**Time**	**Mean (SD)**	**EMM**	**Mean (SD)**	**EMM**	***F* (df^#^)**	***p*-value**	**${\text{η}}_{p}^{2}$**
Performance computerized tests	Pre-test	2.9 (1.1)	2.9	2.6 (1.1)	2.6	0.66(1,287)	0.418	0.002
	Post-test	2.9 (1.1)	2.9	2.8 (1.2)	2.7			
Work ability	Pre-test	3.7 (2.4)	3.8	8.8 (1.4)	8.7	12.32(1,299)	0.001	0.040
	Post-test	4.8 (2.5)	4.8	8.8 (1.3)	8.7			
**Return-to-Work Self-Efficacy-19**								
- Meeting job demands	Pre-test	34.0 (18.7)		Not relevant				
	Post-test	39.2 (18.4)						
- Modifying job tasks	Pre-test	29.3 (13.4)		Not relevant				
	Post-test	33.2 (13.2)						
- Communicating needs	Pre-test	36.9 (14.5)		Not relevant				
	Post-test	38.7 (13.6)						
**Subjective Health Complaints Inventory**								
- Pseudoneurology	Pre-test	7.2 (4.0)	7.2	2.1 (2.5)	2.1	12.64(1,285)	<0.001	0.042
	Post-test	5.5 (3.5)	5.5	1.9 (2.5)	2.0			
- Musculoskeletal pain	Pre-test	9.9 (5.0)	9.8	3.4 (3.6)	4.0	6.58(1,285)	0.011	0.023
	Post-test	8.0 (4.5)	7.8	3.0 (3.2)	3.6			
**Theoretically Originated Measure of the Cognitive Activation Theory of Stress**								
- Coping	Pre-test	2.0 (0.6)	2.0	1.7 (0.6)	1.7	0.78(1,287)	0.379	0.003
	Post-test	1.9 (0.6)	1.9	1.6 (0.5)	1.6			
- Hopelessness	Pre-test	9.1 (1.9)	9.1	10.6 (1.8)	10.5	1.61(1,287)	0.206	0.006
	Post-test	9.3 (2.0)	9.4	10.6 (1.9)	10.5			
- Helplessness	Pre-test	9.6 (2.0)	9.6	11.1 (1.7)	11.0	4.36(1,285)	0.038	0.015
	Post-test	10.0 (1.9)	10.1	11.1 (1.7)	10.9			
**Fear Avoidance Beliefs Questionnaire**								
- Work	Pre-test	20.0 (11.5)	19.7	2.9 (5.9)	4.8	0.26(1,255)	0.611	0.001
	Post-test	18.6 (11.6)	18.4	2.4 (5.0)	4.1			
- Physical activity	Pre-test	9.0 (5.9)	8.9	2.5 (4.7)	3.0	3.61(1,259)	0.059	0.014
	Post-test	8.0 (5.9)	7.9	3.4 (5.2)	4.0			
**36-Item Short Form Health Survey**								
- Pain	Pre-test	4.5 (1.1)	4.5	2.6 (1.3)	2.8	7.48(1,287)	0.007	0.025
	Post-test	4.0 (1.1)	4.0	2.6 (1.4)	2.8			
- Pain related to work	Pre-test	3.4 (1.1)	3.4	1.7 (0.9)	1.8	15.33(1,286)	<0.001	0.051
	Post-test	2.7 (1.1)	2.7	1.7 (1.0)	1.8			
**Hospital Anxiety and Depression Scale**								
- Anxiety	Pre-test	8.3 (4.4)	8.3	4.2 (3.7)	4.2	5.89(1,283)	0.016	0.020
	Post-test	7.0 (4.2)	6.9	3.8 (3.4)	4.0			
- Depression	Pre-test	6.4 (3.9)	6.4	2.3 (2.7)	2.3	8.85(1,283)	0.003	0.030
	Post-test	4.7 (3.8)	4.9	2.0 (2.5)	2.2			

**n*, sample size; SD, standard deviation; EMM, estimated marginal means evaluated with covariate in model (Education); df, degrees of freedom; $\eta_{p}^{2}$, partial eta squared. ^#^All participants did not respond to all questionnaire items or single item questions. The multivariate test statistic Wilks’ lambda was used to display the *F* ratio.*

### Correlations

An exploratory correlational analysis was conducted for the rehabilitation group only (Table 6). Results showed that simple reaction time was positively correlated with SHC pseudoneurology, *r*(240) = 0.13, *p* = 0.044, where faster reaction time was associated with fewer SHC. Response latency in the rapid visual information processing task was negatively correlated with work ability, *r*(240) = −0.16, *p* = 0.012, where faster reaction time was associated with higher work ability scores. Response latency in rapid visual information processing was also negatively correlated with the RTWSE-19 factor “modifying job tasks,” *r*(205) = −0.14, *p* = −0.043, where faster reaction time was associated with higher self-efficacy in modifying job tasks. Hits in the rapid visual information processing task was negatively correlated with work ability, *r*(245) = −0.16, *p* = 0.015, where more hits was associated with lower work ability.

**TABLE 6:** Correlations between cognitive, work and health measures using change scores from pre- to post-test in the intervention group only.

	**SRT**	**RVP, latency**	**RVP, hits**
Work ability	–0.03	−0.16^∗^	−0.16^∗^
SHC pseudoneurology	0.13^∗^	0.05	0.04
SHC musculoskeletal pain	0.08	0.02	0.00
TOMCATS helplessness	0.05	–0.03	–0.03
HADS anxiety	0.05	–0.02	0.03
HADS depression	–0.02	0.09	0.07
RTWSE-19 Meeting job demands	–0.05	–0.11	–0.10
RTWSE-19 Modifying job tasks	0.03	−0.14^∗^	–0.12
RTWSE-19 Communicating needs	–0.00	–0.11	–0.09

*SHC, Subjective Health Complaints Inventory; TOMCATS, Theoretically Originated Measure of the Cognitive Activation Theory of Stress; HADS, Hospital Anxiety and Depression Scale; RTWSE-19, Return-to-Work Self-Efficacy-19; SRT, Simple Reaction Time; RVP, Rapid Visual Information Processing; ^∗^*p* < 0.05.*

## Discussion

This study is the first to systematically compare a broad range of cognitive and emotional functions in occupational rehabilitation patients on long-term sick leave with a healthy working control group. The results showed that patients improved more from pre- to post-test in focused and sustained attention compared to the control group. The changes in performance on memory, executive function and emotion did not differ between the two groups. The rehabilitation group also improved more than the control group in work ability, SHC, helplessness, pain, pain related to work, anxiety and depression. Effect sizes for the cognitive, emotional and questionnaire measures were below 0.06, indicating small effects (Cohen, 1988). The results of the present study suggest that occupational rehabilitation may be associated with improvements in cognitive functioning, in addition to, work and health measures and corroborate the findings of a smaller study conducted in the same clinical setting (Johansen et al., 2016). The fact that all patients were given occupational rehabilitation and a healthy control group were twice administered the same measures as the rehabilitation group is a substantial strength of this study, compared to previous studies where patients seemed to receive several non-related interventions (Eskildsen et al., 2015, 2016) or where a healthy control group was recruited twice (Rydmark et al., 2006; Wahlberg et al., 2009).

In the following we chose to elaborate on the attention findings and its clinical implications. However, this is not ruling out the fact that occupational rehabilitation also seem to positively affect memory, executive function and emotion. Similar to the findings in the current study, improvement in sustained attention has also been reported in individuals on sick leave that received workplace interventions for work-related stress and burnout to increase RTW (Österberg et al., 2012), while a cross sectional study reported impaired sustained attention in burnout patients (Van der Linden et al., 2005). Individuals diagnosed with work-related stress and receiving RTW consultations and/or cognitive behavior therapy at an occupational medicine outpatient clinic showed improvement in prospective memory and processing speed compared to controls (Eskildsen et al., 2015, 2016). However, patients did not receive therapy in a systematic manner and even several patients sought private therapy in addition to the occupational therapy offered during the study. This makes the interpretation about the effects of specific therapies on cognitive functioning challenging. In another study, women on sick leave for work-related stress and depression receiving 10 weeks of cognitive group therapy with a focus on work-related challenges showed at 1 year follow up no impairments compared to a healthy control group in attention and working memory (Rydmark et al., 2006; Wahlberg et al., 2009). Before therapy, all 29 women were on full time sick leave while 18 were in work after therapy. A methodological weakness in these studies, as pointed out by Österberg et al. (2012), was the fact that a new control group was recruited at post-test (Wahlberg et al., 2009) instead of administering the same tests to the controls recruited at pre-test (Rydmark et al., 2006). Finally, a group of patients with fatigue and burnout, receiving cognitive therapy did not show greater gains in executive function related to updating, inhibition and switching compared to a control group (Oosterholt et al., 2012). Although the patient groups in the above studies share overlapping work and health characteristics with the patients in the present study, it is challenging to interpret these results because the RTW or healthcare interventions do not seem comparable. However, in a comparable setting, individuals going through occupational rehabilitation and receiving working memory training have been found to improve more in inhibiting prepotent responses, but not spatial working memory, compared to individuals receiving treatment as usual (Aasvik et al., 2017). This may indicate that adding an extra intervention focusing on cognitive training may improve certain functions more than others.

## Clinical Implications

A key component in occupational rehabilitation programs is the cognitive approach (Aasdahl et al., 2018). The programs utilize treatment principles from cognitive behavior therapy, acceptance and commitment therapy, psychoeducation and motivational interviewing. These are evidence-based psychological treatments commonly applied to reduce sickness absence (Salomonsson et al., 2018). The present results support the argument that complex interventions, such as occupational rehabilitation, have an effect on cognitive, work and health measures (Costa-Black, 2013). To claim a direct relationship between cognitive approaches and improvements in cognitive functioning is spurious because the main treatment components also include physical activity and collaboration with the workplace. It is well documented that physical activity has positive effects on cognition (Ratey and Loehr, 2011) and anxiety and depression (Hovland et al., 2013; Kvam et al., 2016), while the effects on reducing symptoms of pain seem small to moderate (Geneen et al., 2017).

Attention, as cognitive function, is implicated in the development and maintenance of pain, anxiety and depression symptoms (Legrain et al., 2009; Yiend, 2010). The finding that improvements in focused attention were associated with a reduction in SHC related to pseudoneurological complaints indicate that attentional mechanisms were influenced by several factors. Moreover, the application of various forms of cognitive training in chronic pain (Baker et al., 2018), depression (Jonassen et al., 2019) and on patients in occupational rehabilitation (Aasvik et al., 2017) has shown promising effects on cognitive functioning and clinical symptoms. The treatment components in occupational rehabilitation do influence cognitive functioning as shown in the present study, making it relevant to assess possible improvements in cognitive functioning. This could also lead to the development of systematic and evidenced based cognitive training components, implemented as new interventions in future programs, where testing for transfer effects to working life seem important.

Lack of attentional resources may affect the ability to stay focused, for example in a complex work environment. Thus, improvements in focused and sustained attention increase our ability to focus on specific work and daily life tasks for a longer time period without being distracted by symptoms and thoughts, colleagues or background noise. Attention is a crucial cognitive function, underlying many cognitive processes such as memory, planning and decision making (Gazzaniga et al., 2002) together with executive control over attention switching (Miyake et al., 2000). Attentional resources are distributed both unconsciously and consciously (Pashler, 1999), and if impaired, will reduce the ability to concentrate, as well as automatically inhibit irrelevant information or noise from entering our mind. It might therefore reinforce attentional bias toward anxiety, depression, and pain. The assumption here is that increased mental resources, i.e., improvements in attention, could also lead to greater self-efficacy, a factor found to be important for RTW (Lagerveld et al., 2017). The association between attention and self-efficacy for “modifying work tasks” gives some support to this argument.

Recently, a stronger focus on the relationship between clinical (e.g., occupational rehabilitation) and cognitive approaches (cognitive testing) has raised the awareness of this often neglected association (Snyder et al., 2015). It is therefore, highly relevant to better understand the cognitive profile of patients and types of cognitive change to improve clinical practice. Not only attention but all cognitive and emotional functions. Bearing in mind the aim of occupational rehabilitation to increase RTW and increase working life performances (Beier and Oswald, 2012; Loisel and Anema, 2013). Another focus to consider is whether the present study population, representing different occupational groups and status, has general or specific attentional challenges when compared to the general population. This may require more refined validation of the cognitive tests because the target groups are also likely to have occupationally related illness/injury status and not traditionally individuals with psychological and pain related disorders in which the current cognitive and emotional tests have been validated on.

Both cognitive behavior treatment components as well as physical activities in the rehabilitation program may influence cognitive, emotional and behavioral changes underpinning improvements in attention. Examples of cognition and emotion are maladaptive coping strategies such as rumination and worry that maintain symptoms of depression and anxiety because individuals fail to shift focus away from inappropriate thoughts (Williams et al., 1997; Joormann and Gotlib, 2010). For example, the usage of principles based on cognitive therapy may have resulted in improvements in attention and a change to positive coping expectation and cognitive reassessment of the situation, i.e., reappraisal (Ursin and Eriksen, 2004; Joormann and Gotlib, 2010). Related to behavior are low levels of daily life activity resulting in inappropriate coping strategies, that is, cognitive changes, leading to depression and anxiety (Stubbs et al., 2018; Vancampfort et al., 2018). Behavioral activation and adapted training focusing on fear avoidance during physical activity and work situations, may have contributed to attentional improvements and better coping. These changes in attention and coping strategies, related to cognition and behavior, are associated with positive affect, increased quality of life and better functioning in everyday life (Gross and John, 2003). The associations between improvements in attention, better work ability and reduction in pseudoneurological complaints support this line of argument. Here, we focus on how attention is linked to the behavior treatment components without dismissing the link to memory, executive function and emotion. This indicates a relationship between cognitive, work and health measures based on the knowledge that work ability in sick-listed workers has been found to be negatively associated with musculoskeletal pain (Rashid et al., 2018) and co-morbid health complaints (Kamaleri et al., 2009). With the clinical implications in mind it is highly recommended that clinicians are aware of how attentional functions change during a life course perspective and also depending on different contexts.

## Strengths and Limitations

The strengths of this study include recruitment of a large sample of patients and comparing pre- and post-test performances to a control group on several aspects of cognition including attention, memory, executive function and emotion. This is believed to be one of the first studies to comprehensively assess cognitive and emotional functions in occupational rehabilitation patients. The diagnostic profile of the patients was mainly related to depression, anxiety, musculoskeletal pain, and comorbid symptoms. Patients exhibiting such profiles have commonly been subjected to cognitive testing with the added factor that the present sample had RTW as the main aim.

Limitations include the recruitment of individuals from both inpatient and outpatient occupational rehabilitation. This could have introduced a bias in the results due to different criteria used to assign individuals to inpatient versus outpatient rehabilitation. On the contrary, the rehabilitation programs for inpatient versus outpatient do not differ in content, however, the outpatient programs may be experienced as less intense compared to inpatient, which may have implications for the construct of attention assessed in the current study. Five cognitive measures were transformed because the assumptions of homogeneity of variance and normality were violated. Since, three of the five transformed measures were from the attention function, with one measure each from memory and executive function, it could be argued that the transformations were not equally distributed across different functions. However, all transformed variables were related to reaction and response latencies and it is not uncommon to transform these variables given the large variance related to performances on such tasks (Baayen and Milin, 2010). A number of differences between the two groups were not controlled for at baseline and could have affected the results. Factors such as medication, circadian rhythms, motivation and group and individual activities in rehabilitation may have affected cognitive and emotional functioning (Lezak et al., 2012).

## Conclusion

This study has generated new knowledge about changes in focused and sustained attention during occupational rehabilitation. We assume that cognitive behavioral treatment components, physical activity and collaboration with the workplace affect cognitive, work and health measures. The elucidation of greater gains in focused and sustained attention compared to memory, executive function and emotion may benefit clinical practice, such as increasing the awareness to which degree the treatment components affect cognitive and emotional functions differently. This may enhance clinical practice because tailoring specific interventions further may result in greater improvements in all cognitive and emotional functions. Thus, it is recommended that cognitive and emotional functioning is systematically assessed in clinics offering interdisciplinary occupational rehabilitation, similar to physical activity, raising the quality of assessments and enabling individually tailored rehabilitation when baseline cognitive and emotional functioning is taken into account. This may require more refined measures of not only attention, but also memory, executive function and emotion, to elucidate how individuals respond to rehabilitation, time to RTW and to which degree greater or lesser focus should be on various cognitive and emotion related interventions during rehabilitation. This study is a step in the direction toward documenting which cognitive, emotional, work and health changes occur during rehabilitation, where the aim is to tailor treatment components to maximize the benefits for all rehabilitation individuals to increase the chances of RTW.

## Data Availability Statement

The datasets generated for this study are available on request to the corresponding author.

## Ethics Statement

This study involving human participants were reviewed and approved by the South-East Regional Committee for Medical and Health Research Ethics (2013/1559). All participants provided written informed consent prior to participation, and all procedures were conducted according to the Helsinki declaration.

## Author Contributions

TJ wrote the manuscript. TJ, CJ, and IØ performed the statistical analysis. TJ, CJ, HE, PL, WD, and IØ contributed to the conception and design of the study. TJ, IH, and HJ performed the assessments and data collection. All authors contributed to the interpretation of the data and critically revised the manuscript on several occasions.

## Disclosure

All review comments and conclusions are those of the reviewer SP and do not reflect any policy or determination of the Centers for Disease Control and Prevention or the National Institute for Occupational Safety and Health.

## Conflict of Interest

The authors declare that the research was conducted in the absence of any commercial or financial relationships that could be construed as a potential conflict of interest.
